# Validation of modified radio-frequency identification tag firmware, using an equine population case study

**DOI:** 10.1371/journal.pone.0210148

**Published:** 2019-01-09

**Authors:** Rachael M. Milwid, Terri L. O’Sullivan, Zvonimir Poljak, Marek Laskowski, Amy L. Greer

**Affiliations:** 1 Department of Population Medicine, University of Guelph, Guelph, ON, Canada; 2 Department of Mathematics and Statistics, York University, Toronto, ON, Canada; Atlantic Veterinary College, CANADA

## Abstract

**Background:**

Contact networks can be used to assess disease spread potential within a population. However, the data required to generate the networks can be challenging to collect. One method of collecting this type of data is by using radio-frequency identification (RFID) tags. The OpenBeacon RFID system generally consists of tags and readers. Communicating tags should be within 10m of the readers, which are powered by an external power source. The readers are challenging to implement in agricultural settings due to the lack of a power source and the large area needed to be covered.

**Methods:**

OpenBeacon firmware was modified to use the tag’s onboard flash memory for data storage. The tags were deployed within an equine facility for a 7-day period. Tags were attached to the horses’ halters, worn by facility staff, and placed in strategic locations around the facility to monitor which participants had contact with the specified locations during the study period. When the tags came within 2m of each other, they recorded the contact event participant IDs, and start and end times. At the end of the study period, the data were downloaded to a computer and analyzed using network analysis methods.

**Results:**

The resulting networks were plausible given the facility schedule as described in a survey completed by the facility manager. Furthermore, changes in the daily facility operations as described in the survey were reflected in the tag-collected data. In terms of the battery life, 88% of batteries maintained a charge for at least 6 days. Lastly, no consistent trends were evident in the horses’ centrality metrics.

**Discussion:**

This study demonstrates the utility of RFID tags for the collection of equine contact data. Future work should include the collection of contact data from multiple equine facilities to better characterize equine disease spread potential in Ontario.

## Introduction

The 2009 Animal Health Act [1], a document relating to animal health within Ontario, Canada describes 4 health related goals. The first two goals are to: 1) protect animal health, and 2) establish biosecurity measures, i.e. methods that aid in the prevention, control, and response to threats that can affect both human and animal health [1]. Network analysis methods provide an analytical framework that can be used to better inform decisions related to disease prevention, control, and response, specifically in the equine population. Social networks, hereafter referred to as contact networks, describe the relationships between individuals, or groups of individuals in a population [2]. They are often visualized with graphs consisting of nodes and edges. Nodes represent individual entities such as animals or locations, and edges represent the relationships between the nodes [2]. In order to create a contact network, data need to be collected or obtained in such a way that describes a representative portion of the relationships between the individuals in the population of interest. This includes the type of contact, e.g. direct contact such as animal-to-animal contact, as well as the duration and frequency of the contact events. Traditional methods for collecting contact network data include the observation and recording of the contacts that occurred during the observation period, journals or surveys in which research participants document the contact events, and more recently, radio frequency identification (RFID) tags [3,4]. Each data collection method has strengths and weaknesses which can result in different biases (Table 1).

**Table 1:** Summary of strengths and weaknesses of selected contact pattern collection methods.

Method	Strengths	Weaknesses
**Observation**	Less susceptible to error than survey data [5]	Difficult to observe all individuals simultaneously [3]
**Diaries and surveys**	Allows for the specification of the type and frequency of the contact events [4]	Recall and non-response bias [4] Focused on the ego network (a network whose information source is a single node) [6] Costly [4] Often have a small sample size [7]
RFID* **tags**	Relatively inexpensive and easy to use [4] Detect close-range encounters [8]	Technological constraints (e.g. power supply) [2] Radio-technology limitations e.g. radio-wave interference [9] Only provide information about the individuals wearing a tag [4]

* RFID: radio-frequency identification

Although the use of RFID technology for network analysis is relatively new, the technology has a history dating back to World War II. It was used by the British “Identity Friend or Foe system” to distinguish enemy aircraft from their own [10]. The technology has evolved over time, and has since been used in electronic article surveillance (late 1960s), tolling systems (1990s), and for identifying objects with the use of RFID labels such as library books or groceries [10]. The increased accessibility and utility of RFID technology due to its decreasing size and cost have resulted in the implementation of RFID technology in agricultural processes [9]. Agricultural applications include animal identification and traceability, as well as body temperature and behaviour tracking [9,11]. Monitoring of the above characteristics enables the early identification of unusual traits and behaviours which can be used for the advanced detection of disease [12]. Agriculture related RFID applications have been implemented in populations such as finishing pigs [11], free-range broiler chickens [13], and cattle [14].

An RFID system generally consists of two primary components: 1) a reader, and 2) a tag which transmits a signal to the reader. The RFID tags can be described based on the tag’s memory storage capabilities, and the tag’s power source. In the first categorization scheme, the tags are identified as read-only or read-write tags. Read-only RFID tags have no data storage capabilities. Instead, they contain a unique ID which is associated with a database containing information about the object. The read-write RFID tag has a memory source and is able to record data about the object [10].

The second type of classification system categorizes the tags based on their power source. Passive tags are activated by an electromagnetic field generated by the RFID reader. In this case, the tags are powered when they are in range of the electromagnetic field, and can subsequently send data to the reader. Passive tags often use frequencies of 128 kHz, 13.6 MHz, 915 MHz, or 2.45 GHz [15]. Active tags, such as the tags used in this study, are powered by tag-integrated batteries and are able to emit a stronger signal than passive tags. Active tags use radio frequencies such as 455 MHz, 2.45 GHz, or 5.8 GHz [10,15]. The tags used in this study operate on 2.4 GHz frequencies. Although the high frequencies used by active tags allow for a longer read range between the tags and reader, as well as an increased reading rate, the tags require a direct line of sight with the readers and tend to be more expensive [16]. The required frequency for the RFID tags depends on the characteristics of the intended application, such as the read range and the memory requirements [15]. Acceptable frequency ranges are determined by governmental agencies, and therefore differ by location [16].

The use of RFID technology for research purposes has been applied to many disciplines including but not limited to: medicine, engineering, construction, and agriculture [10]. This paper focuses on the use of RFID technology to collect contact data for network quantification and analysis in an agricultural setting. Specifically, the objective of this work was to modify the traditional OpenBeacon tag firmware [17,18] to utilize the onboard tag storage capability. Furthermore, given the type and quantity of data collected with the tags, a secondary objective was to use both traditional network analysis methods, as well as non-traditional network analysis methods to describe the networks. In particular, the networks were characterized by: a) assessing whether the networks were representative of homogenous mixing, b) computing selected node-level centrality measures, and c) assessing the predictability of the networks using survey-acquired information. Given these study objectives, the study hypothesis was that the modified firmware would provide a feasible data collection method for large, agricultural areas. In addition, the quality and quantity of the collected data would enable a comprehensive analysis and description of the time varying contact structure within the equine facility.

## Methods

### System validation procedure

OpenBeacon RFID tags (Bitmanufactory Ltd., Cambridge, United Kingdom [19]) were used to record the contacts that occurred within an equine facility. A contact was defined as an interaction between tags that were within 2m of each other, however, the detection distance can range between 0.5–6.0m (see [8,18,19] for a description of the detection range and accuracy). A distance of 2m was chosen, because it was felt to represent contacts between nodes (horses) in the network that would be considered effective contacts for the possible transmission of an equine respiratory pathogen, e.g. Equine Influenza Virus (EIV), or *Streptococcus equi* also known as the causative agent of “strangles”. While equine influenza (EI) can be transmitted over longer distances, for example, by aerosol, wind, and direct contact [20,21], strangles is transmitted through either fomites or respiratory droplets [22–24]. To account for both pathogen profiles, we considered an effective contact to occur when individuals came within 2 m of each other.

The study took place on an equine facility in southwestern Ontario, Canada over a 7-day period in November 2016. The landscape within the facility was flat, with short building structures that were predominately built with wood. The average outdoor temperature as measured by the closest weather station (longitude: 80°19`49.098”W, latitude: 43°44`05.088” N) during the study period ranged from -3 to 12.3 degrees Celsius. The minimum temperature was -5.5 degrees Celsius while the maximum temperature was 20.5 degrees Celsius. During the study period, there was 0.0 to 13.6 mm of rain, and 0.0 to 1.0 cm of snow [25].

**Fig 1:**
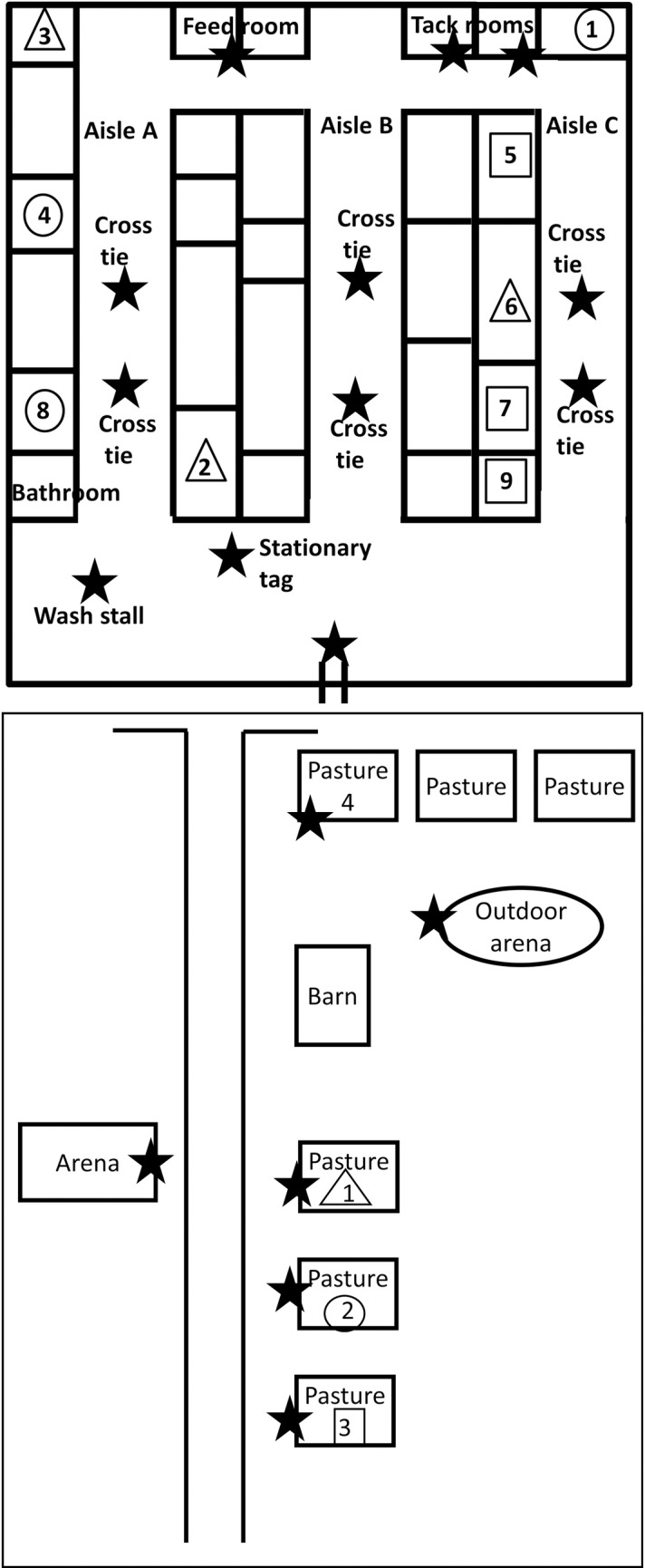
**Map of the barn (top panel) and facility (bottom panel).** The maps are not drawn to scale. With the exception of the barn and the arena, all the locations indicated on the facility map were outdoors. Horses are represented by the numbers in the stall. The shape surrounding the numbers signifies the pasture in which the horse was turned out. The triangle represents pasture 1, the circle represents pasture 2, and the square represents pasture 3. The barn was separated into aisles. The horses were led through the aisles to the barn door in order to exit the barn. The horses were turned out in pastures 1–3, except for on the weekend when at least one horse was moved to pasture 4. Tag locations are indicated with a black star.

This study was reviewed and approved by the University of Guelph’s Research Ethics Board (REB #16AP009) and Animal Care Committee (AUP #3518). The study protocol was explained to the facility manager on November 4, 2016. At this time, recruitment posters and consent forms were posted at the facility inviting all horse owners and employees to participate in this non-invasive study. Written informed consent was obtained from all participants in accordance with the REB (REB #16AP009). The facility boarded 9 horses, 4 of whom were used for lessons. The remaining 5 horses were used for dressage competition and field hunting. All 9 resident horses were enrolled in the study for the duration of the study period. The horses resided in a single building, hereafter referred to as a “barn”. Horses were trained and exercised in both outdoor and indoor training areas (Fig 1).

The tags were placed in a protective plastic cover (Avery Products Corporation, Whitby, Ontario, Canada) which was sealed closed with duct tape (Logix Insulated Concrete Forms, Port Hope and Cobourg, Ontario, Canada). In order to maximize the likelihood that the horse-to-horse contacts would be recorded, the tags were attached to the nose piece of the horses’ halters with vet wrap (3M, United States). The tags are unable to send or receive transmissions through body masses (e.g. horse skull) [8,18]. Therefore, placing the tags on the nose piece of the halter enabled transmissions between horses standing side by side. In addition, the placement of tags on the nose piece meant that close proximity nose-to-nose contacts (e.g. contact sufficient to transmit a respiratory pathogen) would be recorded.

The horses’ halters were kept with the assigned animal at all times. However, since horses are known to be curious animals [26], in order to ensure that the horses did not accidently ingest a tag, the halters were removed from the horses and kept on a horse-inaccessible hook on the pasture fence when the horses were turned-out in the pasture. The horses’ regular daily schedules were carried out as usual.

In addition to the horses, six people volunteered to participate in the study and included: barn workers, horse trainers, and other veterinary health care providers. All human participants wore the RFID tags on lanyards during shift hours. Outside of shift hours, the tags were stored in a central location outside the study area with an additional “bookkeeping” tag. All contact events that occurred within the vicinity of the bookkeeping tag were erased during the data-cleaning phase of the study. Additional tags were placed around the facility at 17 static locations, including the barn doors, indoor riding arena, wash and grooming stalls, and cross ties (Fig 1).

A survey completed by the facility manager captured information regarding each horse’s stall and pasture location, as well as their primary use, e.g. training, competition discipline, etc, and schedule. Briefly, horses were turned-out in their pastures between 8:30 am-4:30 pm. They were fed before being turned-out, and were trained throughout the daytime and evening hours. The horses were turned out in 1 of 3 pastures during the week (Fig 1). An additional pasture was used on the weekend. After turnout, the horses were returned to their stalls, where they remained over-night.

At the end of the study period, a questionnaire was completed by the facility manager. The questionnaire addressed issues such as: the study compliance difficulty, safety with respect to both the human and equine participants, and the invasiveness of the study on the day to day running of the facility.

### Traditional OpenBeacon RFID system

**Fig 2:**
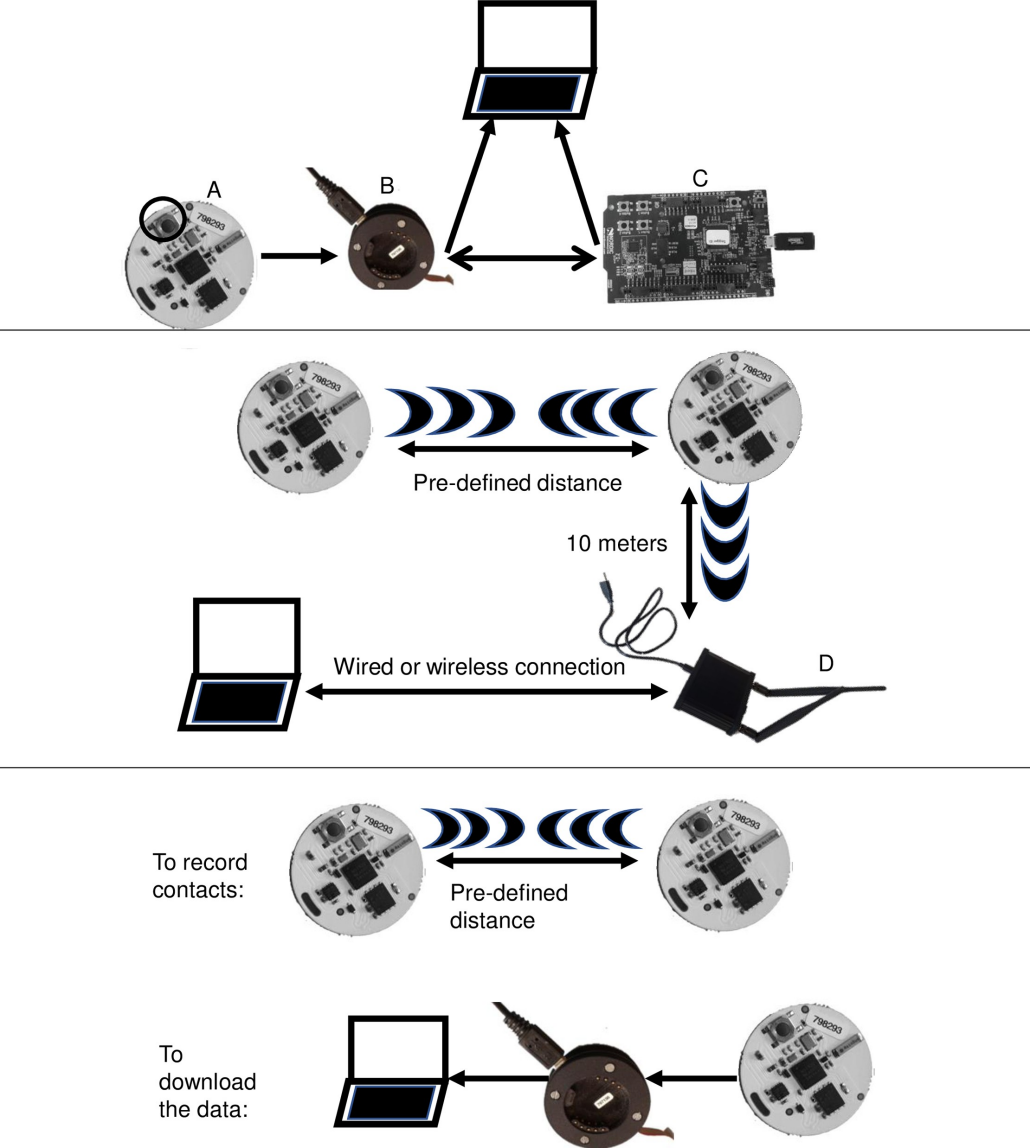
The RFID tag system and components. The top panel shows the components used to flash the tags. The components required to flash a tag are: a computer, a tag (labelled A), a programmer-device (labelled B), and a Nordic board (labelled C). The middle panel shows the traditional RFID setup. In addition to a computer and tag, the communicating tags must be within 10 m of the reader (labelled D) in order to transmit the data to the computer. The bottom panel depicts the modified system, in which the tags operate independently of the computer (top line). The data is downloaded to the computer with the use of the programmer-device. The button on the tag is indicated with a black circle in the top panel.

A typical OpenBeacon deployment protocol is represented in Fig 2. The setup includes active RFID tags that, when they come within a pre-defined distance of each other (e.g. 2 m), exchange signals in a peer-to-peer fashion. If the communicating tags are within 10 m of a reader, the information exchanged is passed through the reader to a connected computer where the data can be stored.

The traditional deployment setup presents some challenges when implementing the infrastructure within agricultural settings. First, while the OpenBeacon tags contain built-in memory, the memory is not used to store data in the default configuration, creating a need for many RFID readers. Second, in order for the communicating tags to transmit the data to a computer, the tags must be within 10 meters of the reader which must be connected to a power source. While this is easily accomplished in indoor settings where power sources are readily available, it is more challenging in outdoor areas such as pastures or fields due to the large areas that would need to be covered, and the associated perceptions of barn fire risks and/or tripping hazards for animals or people. Furthermore, a large agricultural setting or facility would require the use of many strategically placed readers which could result in some contacts not being recorded, as they might occur out of range of the readers. Lastly, agricultural settings may present more extreme variations in environmental conditions such as lower temperatures and precipitation, which can impact the operation and maintenance of the system.

### Modified active RFID tags

To increase the utility of using the RFID tags in a farm (agricultural) setting, the tag firmware was modified to use the tag’s 8 MB flash memory (Adesto Technologies, Sunnyvale, California, United States). This eliminated the need to transfer the data directly to a nearby RFID reader connected to a computer. The tags were 30 millimeters in diameter and operated on 2.4 GHz frequencies. Each tag contained both an internal identification number (ID) stored as a 32-bit unsigned integer, as well as an external ID sticker. When tags came within 2 m of each other, the tags recorded the internal ID of the interacting tags. The process was repeated when the tags stopped communicating (when the distance between the tags increased to more than 2 m), indicating the end of the contact event. A contact event was considered terminated when the tags were out of communication range for 30 seconds. Every contact event that occurred used 16 bytes of memory on each of the tags involved in the interaction. Since the tags have 8MB of flash memory, then given an energy source with sufficient power to store many interactions over long periods of time, the tags can store up to 524,288 interactions, making them suitable for longer study periods with many participants.

The following sections provide details on the tag characteristics, methods of use, and the data storage and processing modifications.

### Tag specifications

The incorporation of an RFID reader in the traditional setup provides the ability to assign a date and time to each individual contact event. While this time-keeping mechanism is ideal, the absence of a reader in agricultural settings makes time keeping more challenging. Individual tags are not manufactured with a real-time clock (RTC), making it impossible to record the actual time at which each contact event starts and ends. To overcome this challenge, the tags were programmed to include a clock substitute that started with the insertion of the coin battery (CR2032). The battery is inserted into the battery cell holder on the back of the tag at the beginning of the deployment period. A light-emitting diode (LED) lights up when the battery is inserted correctly. The faster the initial battery insertion process is completed, the more reliable the time synchronicity between the tags will be since the time at which the clock starts keeping time is dictated by the tags receiving power. Consequently, since the recorded time for a contact event will be slightly different from the standpoint of each of the participating tags, each tag records the contact event start and end times. The time differential between the tags will depend on the time of the battery insertion for the participating tags.

The clock substitute is specified by a counter that increases by 1 unit per second. Therefore, in the case of battery removal, the counter will resume counting from the last recorded time with the insertion of the new battery. The insertion of the battery also begins the peer-to-peer (a process in which both components have the same functionality, i.e. sending and receiving signals) signal transmission and receiving process. Consequently, the calendar day and time of the battery insertion process should be recorded for use in the post-processing stage.

In the current version of the tag firmware, the tag detection and recording processes occur once every second. While tags can interact with and record data from an unlimited number of tags, only one observed contact with another tag is recorded at a time. For example, when tag A encounters tag B, both tags A and B record this contact. If a third tag (tag C) happened to encounter tag B while tag B was recording the contact event with tag A, the recording action is put on hold while tag B senses the presence of tag C. Once tag B has sensed the presence of tag C, the recording action between A and B will continue. Tag B and C will both record the contact with each other. While this limitation affects the order of operations occurring within the tag (the recorded data will not necessarily be sequential), its effect is mitigated through sorting during the data processing stage where the data is aggregated into 24-hour periods based on the contact event start date.

### Setup

The required software for the tags can be run on Linux and Apple MacOS operating systems. In addition to the firmware code, which is available upon request, a JLINK tool is required and can be downloaded from: https://www.segger.com/downloads/jlink/. The JLINK tool enables communication between a Nordic board and the computer through a USB connection (Fig 2). In addition, the JLINK tool can be used for debugging purposes which is supported by using the JLINK tool in conjunction with the board.

Lastly, a computer is used for scripting and compiling the code. The code can then be loaded onto the tag chip with a compiler. The tag chip uses the ARM compiler which can be downloaded from: https://launchpad.net/gcc-arm-embedded.

The Nordic board and programmer-device (Fig 2) are necessary to flash (update the firmware) the developed firmware to the tags. Tags are flashed by attaching the programmer-device to the board and loading the firmware onto the tags with a series of terminal commands. The developed firmware is housed in a GitHub repository and is freely available to interested research groups upon request. Once each tag has been flashed, the tags can be used to collect the described contact data. The updated firmware contains three different functionalities which can be accessed via a button on the tags (see Fig 2 for the button location). The three functionalities are: transfer the data from the tags to the computer (one button push), check the tag battery status (two button pushes), and clear the tag memory (three button pushes). The tag’s memory needs to be cleared before each subsequent deployment.

Once the tags have been deployed, the tags will continue to send signals, receive signals, and record the data until the battery is either removed or is depleted of power. Once the battery has been removed, the tags can be connected to a computer via the programmer-device and the data can be downloaded for processing.

After the data has been downloaded, the tag memory can be cleared in order to prepare the tags for the next deployment. The tag memory can be cleared either with or without the tag programmer-device; however, the process is power intensive and uses substantial amounts of battery power. Therefore, it is advised to power the tags with the programmer-device and a connected computer which provides an unlimited energy source during the memory erasure process. The memory erasure process can take up to 200 seconds, with an average of 80 seconds.

### Data storage and analysis

The data is transferred from the tags to a computer where it is stored in a MySQL (Oracle Corporation, Redwood Shores, California) database. The following section describes the data cleaning and processing stages.

The database is composed of multiple tables which contain information about: the trial number, each of the participating tag IDs (i.e. a source tag (the tag from which the data is being accessed) and a partner tag (the tag that came in contact with the source tag)), the time of the contact event for both the source tag and the partner tag, and a number denoting the status of the contact event, where the status is one of: contact event begins, contact event is ongoing, or contact event ends. The time stamp from the source tag is used to calculate the duration of each contact event. The contact start time is subtracted from the end time in order to obtain the contact duration.

A list mapping the external ID to the internal ID of each of the tags was entered into a tag database prior to the tag deployment. The collected data is subsequently compared to this list in order to ensure that there are no aberrant data. Further data filtering is accomplished by entering the study deployment start and end dates and times. Any data that falls outside of this range (i.e. the study period) are discarded during the processing step. This processing step is important as it allows the researchers to remove the tag batteries at a convenient time and place, as opposed to at the original deployment location (e.g. in the case of inclement weather during the completion of the deployment).

Finally, the recorded contact events that occurred in the vicinity of the bookkeeping tag (that was used to track tags that were not being used) were deleted. The data were aggregated by 24-hour periods (the first and last day of the study were less than 24 hours due to the deployment setup and clean up time). Data outputs include seven comma separated value (CSV) files (one for each day of the deployment). The CSV files contain the internal ID of the participating tags in each contact event on each respective day, as well as the “weight” of the contact event which represents the total contact duration for each pair of participants throughout the 24-hour period.

The remainder of this paper will focus on validating the collected data during this test deployment with regard to horse-horse, horse-person, and horse-location contacts. The empirical data was validated against the expected contact patterns as described in the facility survey. A network analysis was completed on the horse-horse networks using R version 3.3.0 [27], specifically, with the igraph package [28].

### Data analysis

Disease transmission models are often used to evaluate the expected impact of disease prevention and control strategies in a population. The models often rely on the assumption of homogenous mixing with each individual having an equal probability of coming in contact with any other individual in the population. While this assumption is convenient, it is not appropriate for all populations, such as agricultural populations which tend to be highly structured resulting in non-random contacts. Therefore, heat maps were used to assess the conformity of each of the contact networks to the assumption of homogenous mixing. The assumption of homogenous mixing was evaluated for both the day-long networks, as well as a combined week-long network.

A node’s importance in the network can be examined using a variety of centrality measures. Each centrality measure indicates the node’s influence on the network with respect to a different network measure. Specifically, the current study used unweighted and weighted degree, and eigenvector centrality to assess the node-level significance on the network. These measures were chosen based on their ease of applicability to the transmission of diseases through the facility. The unweighted degree represents the total number of unique contacts a horse had with other horses and was calculated by summing the number of edges incident to the respective node [29]. Therefore, the degree can range from 0 to *N*−1, where *N* represents the total number of nodes in the network.

The weighted degree represents the total duration of contact (hours) a horse had with all other horses in the network on each day of the study. The weighted degree, hereafter referred to as “strength”, was calculated by summing the contact duration, representing the edge weight, for each contact event that a horse of interest had with other horses in the population [30]. The minimum possible strength is 0 (for an isolated horse), while the maximum possible strength varies based on the number of contacts each node had with other nodes, and the duration of each contact. Therefore, the maximum possible strength can surpass 24 hours, as a horse may come in contact with many other horses for long periods of time.

Lastly, the eigenvector centrality is similar to the degree, however, it takes into account the adjacent node’s centrality, thus presenting a more comprehensive idea of second degree contacts a horse may have [31]. The eigenvector centrality can range from 0 to 1, where a higher value indicates a more connected node.

The facility survey was used to assess the possibility of predicting the contact patterns without the use of RFID tags. The information regarding each horses’ stall and pasture location was used to create a network (hereafter referred to as a “survey-based network”) in which it was assumed that all horses that shared a pasture or barn came in contact with each other. In the event that a horse was moved between pastures during the study week, the pasture in which the horse was turned out for the majority of the week was used in generating the survey-based network. A classification table was used to compare the survey-based network to a weeklong network created by combining the data from each study day. Specifically, in the classification table, the unweighted network specified by the data collected with the tags was used as a gold standard, and the unweighted survey-based network was used as a predictive test. The classification table was used to calculate the sensitivity (sn), specificity (sp), and positive and negative predictive values (PPV and NPV respectively) of the static network. The sn represented the ability of the unweighted, survey-based network to correctly predict the occurrence of contacts recorded with the RFID tags. Similarly, the sp represented the ability of the survey based-network to identify the absence of contacts as described in the empirical networks. The PPV and NPV represent the probability, that given that survey-based network recorded the presence or absence of a contact, the tags also recorded the presence or absence of a contact.

## Results

### Technical results

Of the 33 tags deployed during the study period, 22 of the tags recorded at least 6.9 days worth of data. In total, 4 out of 33 tags recorded up to 6 days worth of data (two of the tags were attached to the entrance to the farm, so it is likely that no horses left the farm during this period. Unfortunately, this data was not captured in the facility survey.). Out of the 33 batteries, 29 retained their charge until part way through the 6^th^ day of the study meaning that 87.9% of the tags were powered for the majority of the study period.

One of the tags assigned to a veterinary health care professional during the tag deployment was not used, as the individual did not visit the facility during the study period. Given the lack of use, this tag was stored with the bookkeeping tag for the entire duration of the study. Therefore, after the data cleaning process, all data related to the specified tag should have been erased. However, after the data cleaning process, there were still multiple contacts between the unused tag and other tags. This may be due to the tags sporadically restarting, providing gaps of time in which the bookkeeping tag was not communicating with other tags in the vicinity.

### System validation

**Fig 3:**
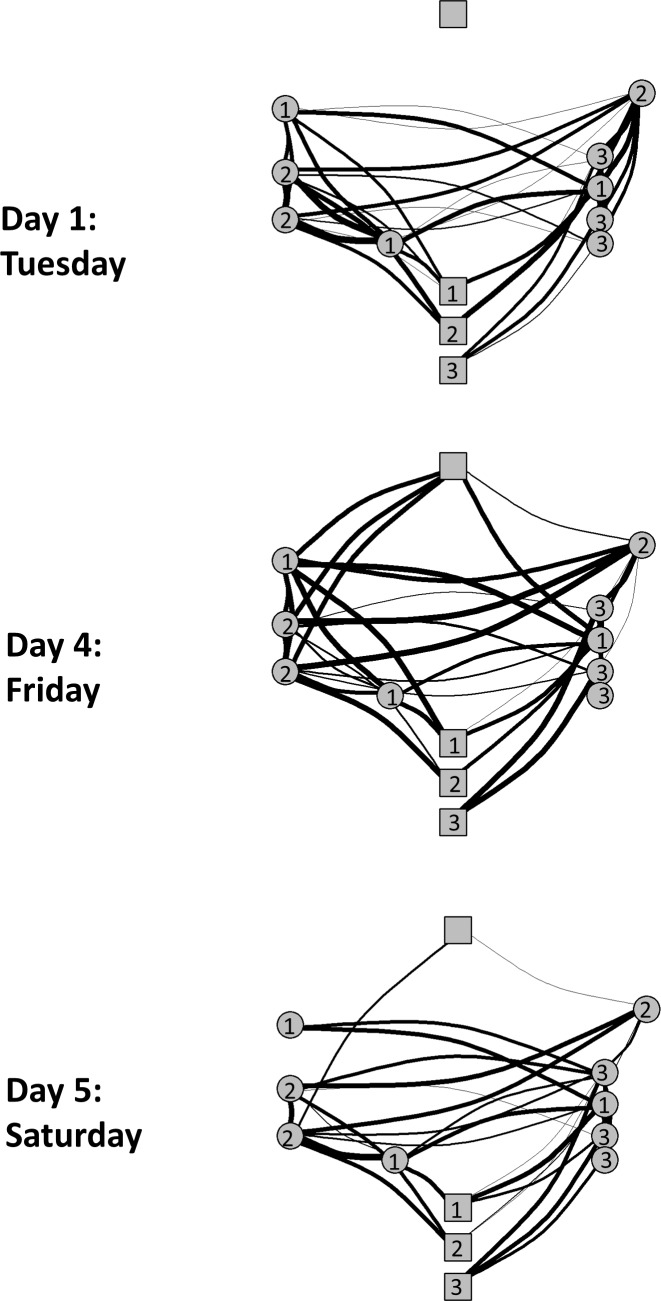
Selected horse and pasture contact networks. Each node in the network corresponds to the geographic placement of the pasture and stall locations on the map in Fig 1. The horses are represented by circular nodes and the pastures are represented by square nodes. The number in each node representing a horse is indicative of the horses’ pasture and corresponds to the number in each the pasture node. The network in the top panel represents the contacts that occurred on day 1 of the study, the network in the middle panel represents the contacts that occurred on day 4 of the study, and the network in the bottom panel represents contacts that occurred on day 5 (Saturday) of the study. The thickness of the edges between each pair of nodes represents the contact duration for the respective day. Therefore, a larger edge represents a longer contact duration.

Sample networks from selected study days can be found in Fig 3. The networks represent contacts between horses and pastures. The top panel shows the network of contacts that occurred on day 1 of the study, when the horses were turned out in one of three pastures. The middle and bottom panels show the contacts that occurred on days 4 and 5 of the study, when the horses came in contact with an additional pasture. The horse-pasture contact duration for each of these days is summarized in S1 Table. Additionally, the duration of the horse-horse contact for days 1 and 3 of the study is summarized in Table 2. Horses whose stalls were in the same vicinity in the barn tended to have a longer duration of contact than other horses.

**Table 2:** Contact duration (hours) between horses on selected study days. The cells below the diagonal represent contacts that occurred on day 1 of the study, while the cells above the diagonal represent contacts that occurred on day 3 of the study. Highlighted cells indicate horses whose stalls were in the same area in the barn (Fig 1). All of the contact durations on the bottom half of the table represent the contacts that occurred on day 1 (Tuesday) of the study, while the contacts above the diagonal represent contacts that occurred on day 4 of the study (Thursday). The contact duration between the horses that were in the same area in the barn tended to be longer in than between the other horses.

Contact event duration (hours)		Horse								
		1	2	3	4	5	6	7	8	9
**Horse**	**1**			0.05	6.11	0.67	<0.00	0.01	0.89	
	**2**	<0.00		5.63	0.23		4.12	<0.00	5.73	
	**3**	<0.00	0.69		0.17		6.24			
	**4**	0.24	11.89	0.31			<0.00		21.76	
	**5**	2.82	<0.00	<0.00			0.23	1.31		5.44
	**6**	2.18	1.31	0.46		0.11		0.15	<0.00	0.02
	**7**	0.23				0.24	15.04			13.92
	**8**	0.23	15.75	0.19	15.92		<0.00			
	**9**	0.13			0.01	2.30	3.82	15.58	<0.00	

**Fig 4:**
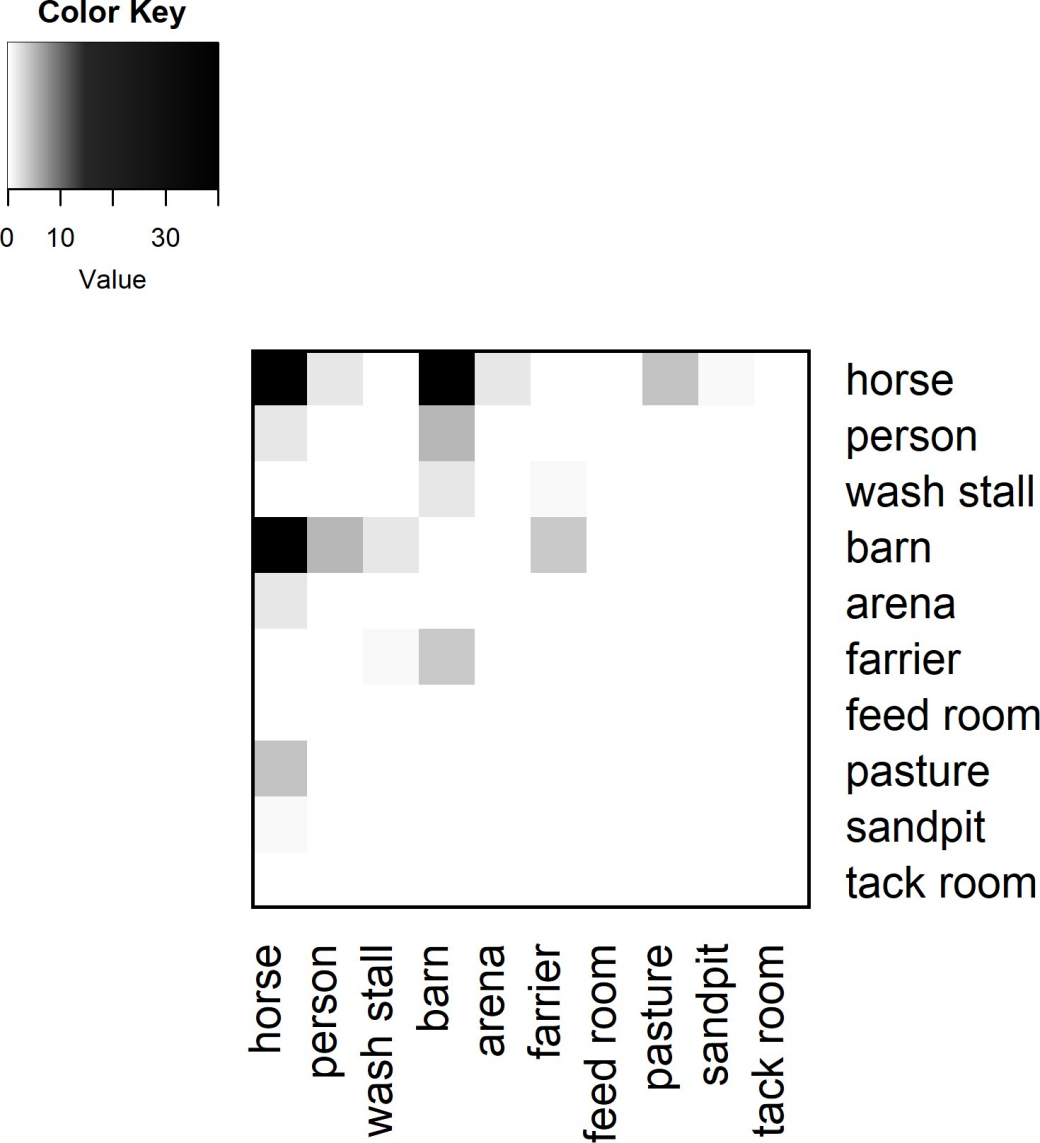
Sample heat map of aggregated tag contacts. The heat maps show the amount of contact that tags (based on individual tag assignment) had with each other. Darker colours indicate a higher duration of contact (hours) throughout the day.

The facility survey did not state which horses were moved to the fourth pasture for which days, rather, it was stated that a horse moved to a fourth pasture on the weekend. Fig 4 and S1 Table imply that this move occurred on day 4 (Friday) of the study. Given the short contact duration between the horses and pasture 4 on day 5 of the study, it is likely that the contact occurred when the horses walked past the tag activating it (pasture 4 is located outside the barn door). Regardless, the tag recorded additional contacts on these days, as was expected by the knowledge gained from the survey.

The contact data collected corresponds to the data that one would expect due to the facility structure and scheduling (these additional details were collected using the survey). The networks in Fig 3 indicate that tags located on the horses’ halters had an increased amount of contact (indicated with a thicker line) with other horse tags that shared a pasture or barn aisle. This corresponds to the large amount of time spent in the pastures (8:20 am -4:30 pm) and stalls. This trend is repeated daily, owing to the daily similarities in the facility schedule, and further validating the functionality of the tag’s sensing and recording abilities.

Lastly, while most of the data appears to make sense in terms of the facility schedule, there are some anomalies. For example, Fig 4 shows a heat map of the aggregated weights for each type of tag location (e.g. horse, barn, arena etc.). In other words, the heat map examines the total duration of contact between, for example, all horses and all pastures. From the figure, it is clear that at least one tag in the barn came in contact with the tag in the wash stall, both of which were fixed to the facility walls. Neither tag was within 2 m of each other during the deployment. This is the only inconsistency of its kind, as the remainder of the recorded contact events appear to be plausible given the facility’s daily schedule and the expected contacts. Additionally, although the tags recorded contacts that were expected to occur, the contact duration recorded was sometimes less than expected (Table 2). This can be caused by various factors including the inherent limitations due to radio-waves, or the fact that the tags may have sporadically restarted. Interference, whether constructive or destructive is a common occurrence and is to be expected in radio transmission systems [32].

In general, over the course of the study period, horses had the most contact with each other (97.04 hours over the course of the study period), followed by horses and the barn (82.18 hours over the course of the study period; this includes the barn door and cross ties). People came in contact with most areas in the facility, however, they had minimal contact with the pastures, tack room, and feed room (0.04, 0.01, and 0.01 hours). This is likely due to the fact that the contact between people and the pastures, tack room, and feed rooms were only recorded when they entered and exited these locations.

### Network description

#### The assumption of homogenous mixing

**Fig 5:**
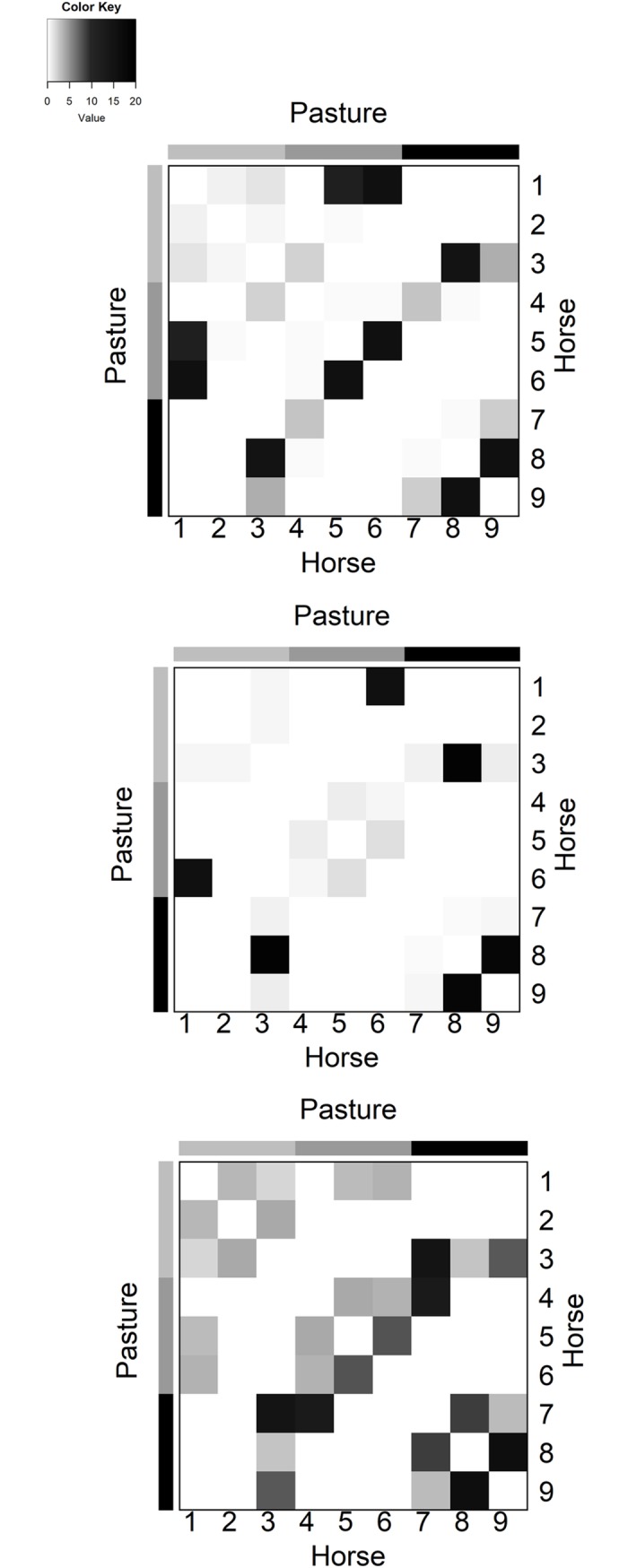
Summary study results. Heat maps of the duration of contact (hours) throughout the day. Horses are numbered in conjunction with the numbering system in Fig 1. Only three days of results are shown (day 1 (top row), day 5 (middle row), and day 7 (bottom row)), as the results were similar for each day of the study. The heat maps are not suggestive of homogenous mixing. The full set of figures can be found in the supplementary information in S1 Fig.

**Fig 6:**
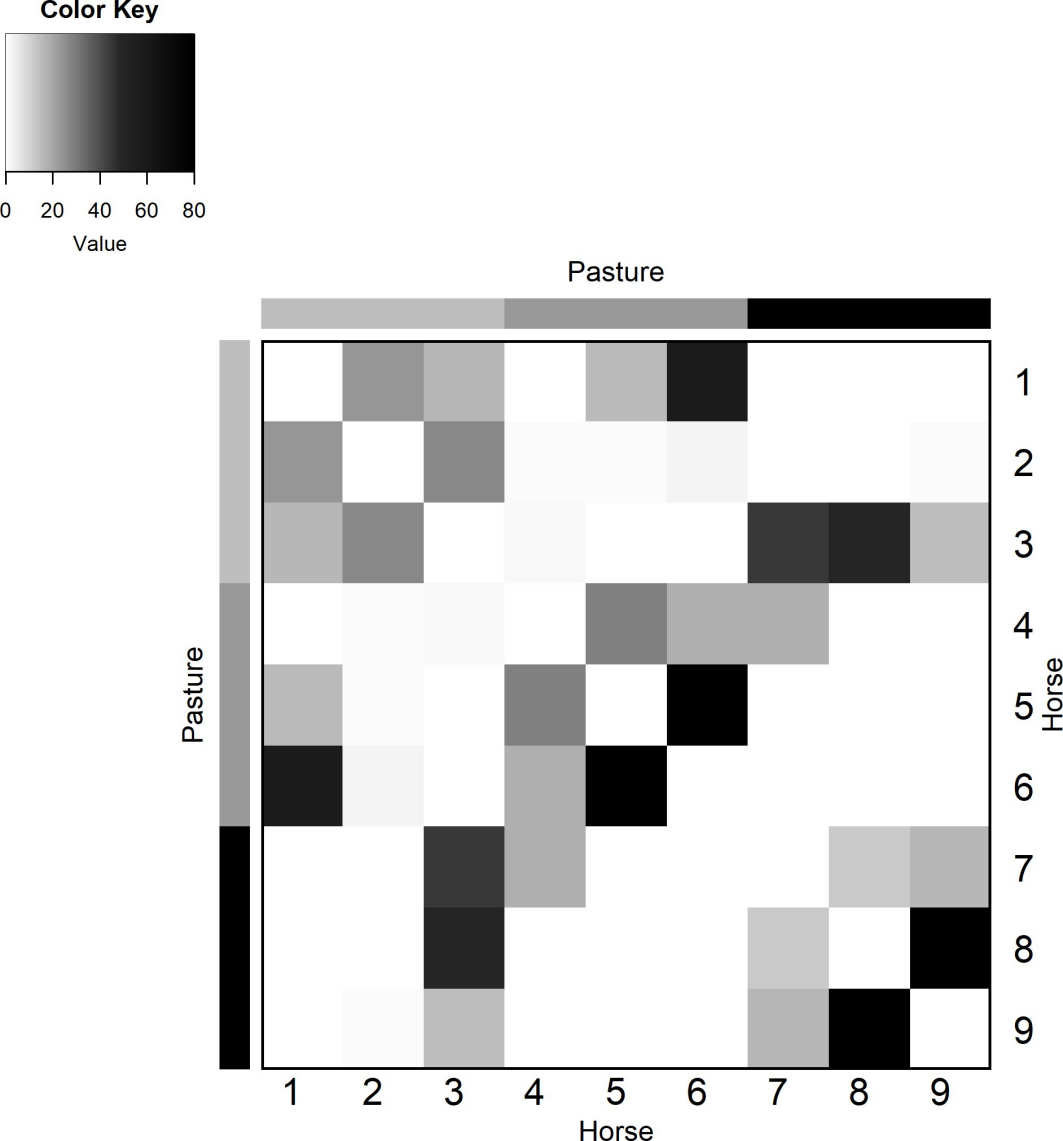
Heat map of the 7 combined dynamic networks. The heat map is not suggestive of homogenous mixing. The colored panels on the left and top axes represent the different clustering of horses by pasture.

The heat maps used to assess the homogenous mixing assumption for each study day indicate that horses did not come in contact with all the other horses over the course of the week (Fig 5). Furthermore, the weeklong static network is similar in shape to each of the individual, day long heat maps (Fig 6). The contact patterns within this facility do not appear to satisfy the assumption of homogenous mixing.

#### Node centrality

**Fig 7:**
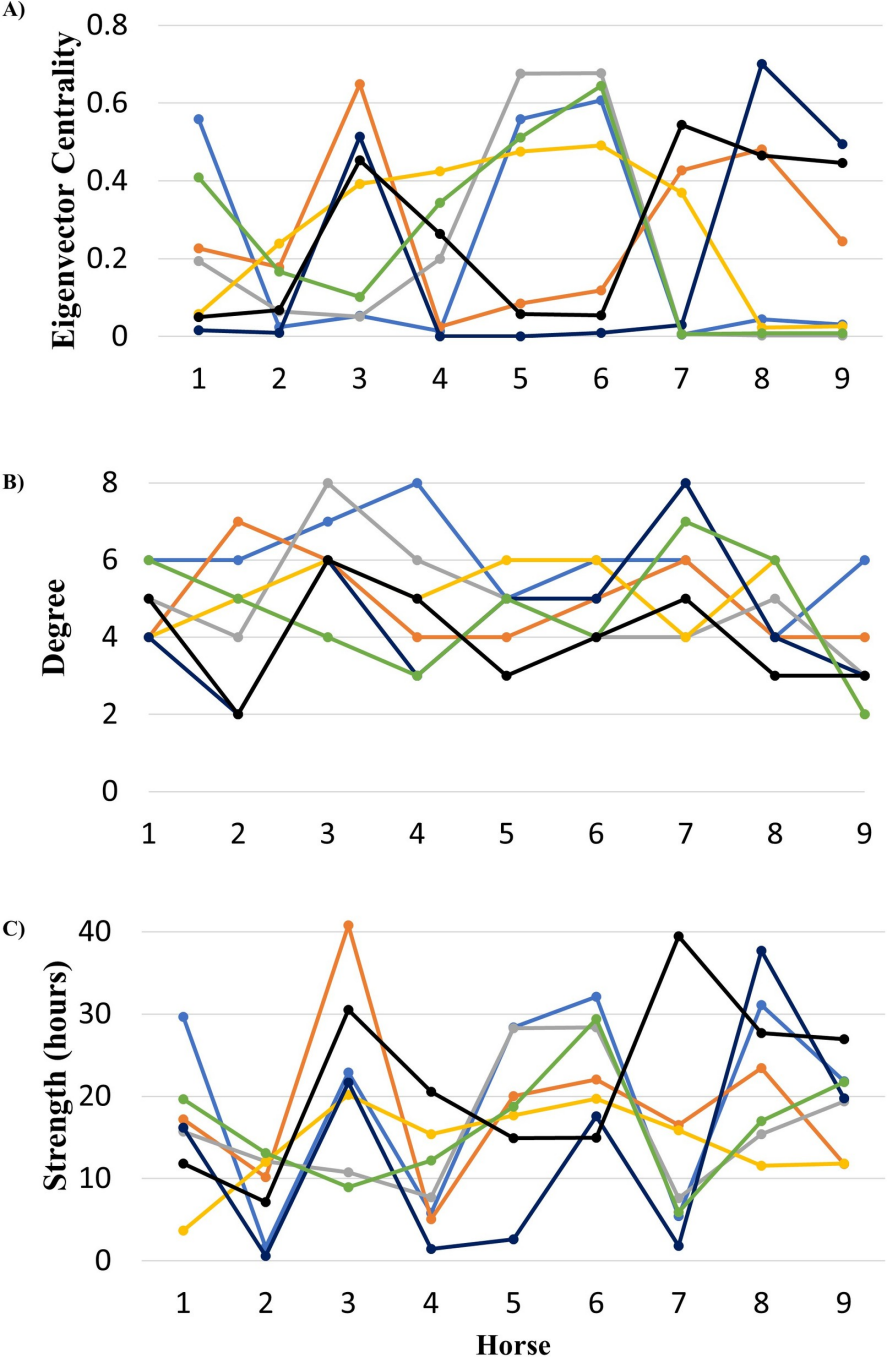
Selected centrality measures for each horse on each study day. Centrality measures include eigenvector centrality (A), degree (B), and strength (C). Each day, a different horse was the most central horse (day 1 = light blue, day 2 = orange, day 3 = grey, day 4 = yellow, day 5 = dark blue, day 6 = green, day 7 = black). The eigenvector centrality ranged between 0.0 and 0.7, while the degree and strength ranged between 1 and 8, and 0 and 40 respectively. No trends were evident in the centrality plots.

The network centrality indicates that the horse with the highest degree varied throughout the study week. The survey results indicated that the horses with the highest degree on each study day differed in terms the horse’s main use (e.g. teaching, pleasure riding etc.) and the aisle in which they were housed overnight. The degree centrality ranged from 2–8 with the average daily degree ranging from 4 to 6 (Fig 7).

The horse with the highest strength and eigenvector centrality also varied over the study period, however, one horse in particular had the highest strength and eigenvector centrality on 4 of the 7 study days. The horse with the highest strength also had the highest eigenvector centrality for each study day. The horse’s strength, ranged from ~0.63 hours to 41 hours. The eigenvector centrality ranged from less than 0.01 to 0.70 (Fig 7).

#### Comparison of the tag-acquired network to the survey-based network

The classification table resulted in a high sensitivity (*sn* = 1) and specificity (*sp* = 0.82) when comparing the survey-based network to the week-long tag-acquired network. The positive and negative predictive values were 0.97 and 1.0 respectively.

When comparing the survey-based network to each day of the tag-acquired network, the classification table resulted in high sensitivities (*sn* = 1 for each study day) and low specificities (*sp* ≤ 0.33) for each study day (Table 3). Furthermore, both the positive predictive values and negative predictive values were relatively high (≥ 0.5 and 1 respectively) (Table 3).

**Table 3:** Classification table output to assess the effectiveness of the survey collected data for predicting contacts. The networks formed with the tag-collected data represents the gold standard and the network formed with information from the survey represents the test. The test sensitivity always correctly predicted the existence of contacts, however, the specificity was relatively low, indicating an inability to correctly predict an absence of contacts. Both the positive and negative predictive values were relatively high (≥ 0.5).

	Day						
	1	2	3	4	5	6	7
**Sensitivity**	1.00	1.00	1.00	1.00	1.00	1.00	1.00
**Specificity**	0.33	0.24	0.24	0.24	0.22	0.23	0.20
**Positive predictive value**	0.75	0.61	0.61	0.61	0.56	0.58	0.50
**Negative predictive value**	1.00	1.00	1.00	1.00	1.00	1.00	1.00

## Discussion

The evolution of RFID technology has provided opportunities to further research in all disciplines, including that of mathematical epidemiology. Although OpenBeacon RFID technology has been used in previous studies to collect contact data [17,33–35], this is the first study to make use of the onboard storage for data collection and to specifically describe the methods and tools developed for this type of research deployment. This adaptation of the traditional setup allows for the extension of the OpenBeacon RFID tags from primarily indoor settings, to outdoor settings, extending the range of potential interdisciplinary research in agricultural and veterinary settings.

Although the network size used in the pilot study was small, it provided the opportunity to test and validate the updated RFID firmware and data processing procedures. Similar contact patterns were seen on each day, corresponding to similarities in the daily farm schedule. While some differences exist in the contact network data collected, these differences are likely due to stochasticity in human behaviour. This stochasticity can impact aspects such as the horse’s daily schedule (e.g. when they are exercised) and contacts (e.g. which other horses are in the vicinity of the cross ties being used).

There were no observable trends with respect to the horse-horse centrality measures, however, it is important to note that the degree centrality ranged between 2 and 8. The degree range is important as it helps to describe the range of outcomes that could potentially arise from the introduction of an infectious disease into the study population [36]. If the infectious horse has a degree of 2, then, at most the infectious horse can infect two other horses, potentially limiting the spread of the disease. Alternately, if the degree of the infectious horse is 8, then there is a higher likelihood of the disease spreading to the entire study population. Thus, both the degree distribution and the position of the initially infected horse in the network have the potential to define the range of outcomes on the network.

A similar outcome can be inferred from the horse-horse eigenvector centrality measure. While the eigenvector centrality ranges from >0.00 - ~ 0.70, if a horse with a higher eigenvector centrality was to become infectious, it could have catastrophic effects on the adjacent horses in the network, which also have relatively high degrees.

The comparison of an unweighted, survey-based network in which each horse came in contact with other horses in its barn, as well as horses with whom it shared a pasture indicated that the survey-based network was able to correctly predict all the contacts that occurred (*sn* = 1) when compared to the unweighted network formed with the tag-collected data. However, the low specificity (*sp* ≤ 0.33) indicates that the survey-based network over-estimated contacts, as it failed to identify contacts that did not occur. The high sensitivity and low specificity are likely due to the barn management style. Since all horses boarded at the facility resided in a single barn, the survey-based network, was, in essence representative of a complete network in which all horses came in contact with each other. Given the complete network structure of the survey-based network, the network would fail to predict or account for the absence of contacts between horses. The complete network structure of the survey-based network led to high negative predictive values (*NPV* = 1) indicating that the survey-based network correctly identified individuals who did not come in contact. Furthermore, the positive predictive values were relatively high indicating that the majority of contacts that did occur were represented in the survey-based network.

While the contact pattern predictability results are important in terms of data inference without the use of tags, they come with the following caveat; the classification table was computed on unweighted networks. Therefore, the results do not provide insight into the duration or frequency of the contact events. If the transmission of a disease is dependent on the duration or frequency of contact events, then the predictability results are inapplicable in the study of contact patterns and their effect on disease transmission. However, if a single contact event is sufficient for the transmission of a pathogen, for example, in the case of highly transmissible infectious diseases, then the predictability results provide a good approximation of the contact rate. This outcome can then be used in a disease transmission model to study realistic outcomes in the presence of various prevention and control strategies without the use of technology such as RFID tags.

With respect to the updated RFID technology, the tags functioned well with some imperfections. The batteries did not survive for the full seven days (some batteries fully discharged within the 6^th^ day). Although this might be partly due to the cold, this problem has persisted in other subsequent studies that were conducted on larger facilities in warmer temperatures (unpublished data). In subsequent studies, faster battery discharge was observed in the tags assigned to human participants. The increased battery discharge can be explained by the fact that these tags were stored together for long periods of time, resulting in an increased frequency of tag-to-tag communication. Furthermore, for unknown reasons, the tags do on some occasions appear to sporadically restart. While this error has the potential to cause a loss of data, the restart takes minimal time, thus minimizing the impact to the data. This discrepancy in the data is further exaggerated by the inability for multithreading and the lack of a real-time clock. However, this is in the order of milliseconds, resulting in minimal data loss. Furthermore, these limitations have helped to identify areas for further research to improve the quality of the collected data.

An analysis of the horse-horse and horse-pasture contact event durations highlighted the impact of the colder temperatures on the tag functionality. While the tags recorded the change in pasture use on day 4 of the study, all of the contact event durations associated with the contact events that occurred when the horses were turned out in their pastures were less than would have been anticipated according to the study survey. However, a comparison of the horse-horse contact given the horses’ locations in the barn resulted in more reasonable contact durations. This discrepancy indicates the suboptimal functionality of the tags in colder temperatures.

Lastly, RFID technology has many inherent advantages and disadvantages. While the tags used in this study did not require a direct line of sight to each other, they are incapable of transmitting signals through body mass [18]. Furthermore, the tags are susceptible to electromagnetic interference due to radio-wave distortion, absorption, and deflection [9,32]. This interference can affect the data in a multitude of ways including the obstruction and degradation of the RFID performance which can result in spurious reads [32]. Due to the nature of radio-wave technology, participating tags might record slightly different contact durations. This can be attributed to different reasons including the tags ability to sense a limited number of tags at a time, and the various forms of natural and artificial interference that can be anticipated in any type of radio transmission. A review of the recorded data from both the source and partner tags resulted in minimal difference between the tags’ output. Therefore, given the large aggregation period of 24 hours, a heuristic was used to consider the original data as collected by a single tag, however, methods of compensating for data inconsistencies include averaging the recorded times from the participating tags, or using the maximum time recorded between the participating tags.

Although limitations exist, it is important to consider them in the context of the study as a whole. First, the data was aggregated by 24-hour periods. Therefore, minor inconsistencies in time have minimal effect on the overall network description. Second, alternate methods of data collection can be time consuming and costly, and may not accurately reflect the entire network. For example, diaries and surveys tend to focus on the ego network (the network of the individuals in question), as opposed to focusing on the relationships within the population as a whole [6]. Further biases include recall and non-response bias. Similarly, data collection by observation has the potential to miss observations and is subject to human error. In comparison to these alternate methods of data collection, the framework for collecting contact data using RFID tags described here has the potential to quantify a more reliable and comprehensive set of data. However, multiple data collection methods can be used to cross validate the data.

Third, the study design had the potential to impact the resulting contact networks. For example, when the horses were in their pasture, the halters and tags were placed on a halter hook on the pasture fence. The data collected during this time would indicate that the horses in the pasture were all within 2 meters of each other. While horses tend to be social creatures, the assumption that the horses congregate while in the pasture might be incorrect, causing the collection of contacts that never occurred. Furthermore, the tags were programmed to include a washout period of 30 seconds. This means that if two previously interacting tags failed to detect each other for 30 consecutive seconds, the tags recorded that the contact event had ended. The premature termination of a contact event has direct implications for the role of contact in the transmission of diseases, both in terms of contact frequency and contact duration. The washout period impacts the duration of the contact event, for example, in the case that the horses changed position and the tags were unable to communicate, cutting the contact event short, and the frequency of the contact events, when, for example, a horse may be temporarily moved interrupting the tag-to-tag contact. In this case, the contact would be counted as two contact events instead of one. However, the overall purpose of the study was to collect contact data that could enable the transmission of respiratory diseases between horses. Therefore, the discrepancy in the recorded contact events due to the tag location on the pasture fence, as well as tag functionality with respect to the washout period has minimal impact on the network for the intended use.

Lastly, RFID tags are becoming increasingly inexpensive and uncomplicated to deploy. The time component of the data collection involves preparing the tags, setting up the deployment, downloading the data, and clearing the tag memory at the completion of the deployment. The post-study questionnaire indicated that the RFID tags resulted in minimal intrusion into the daily activities of the farm. Horses appeared to be unaware of the tags, and from the point of view of the participants, the main difficulty was remembering to wear the tags and to move the horse halter from location to location (if it was not normally done).

Overall, we found the modified tags to provide reliable, biologically plausible data, which would be difficult to obtain using the traditional RFID system deployed in an agricultural setting or by using other methods. The tags provided an easy, safe, non-invasive, and non-intrusive means of collecting data that can be used to further network analysis research in agricultural settings.

## Supporting information

S1 Checklist NC3Rs ARRIVE guidelines checklist. (PDF)

S1 Fig Complete set of heat maps for each day of the study period (panels A-G show the heat maps from days 1–7 respectively). Darker cells represent longer durations (hours) of contact between horses.
(TIF)

S1 Table Contact duration (hours) between horses and pastures on the selected study days. The tag that was located on the fence of pasture 4 was only activated on days 4 and 5 of the study. However, the contact duration on day 5 of the study was negligible, indicating that the tag was activated as horses walked passed the pasture. Many of the contact events were shorter in duration than what would be expected. This can be explained by the cold outdoor temperatures.
(DOCX)
