# Supplementary figures and images for: Validation of modified radio-frequency identification tag firmware, using an equine population case study
Source: PLoS One. 2019 Jan 9;14(1):e0210148. doi: 10.1371/journal.pone.0210148 (PMC6326514; doi:10.1371/journal.pone.0210148)

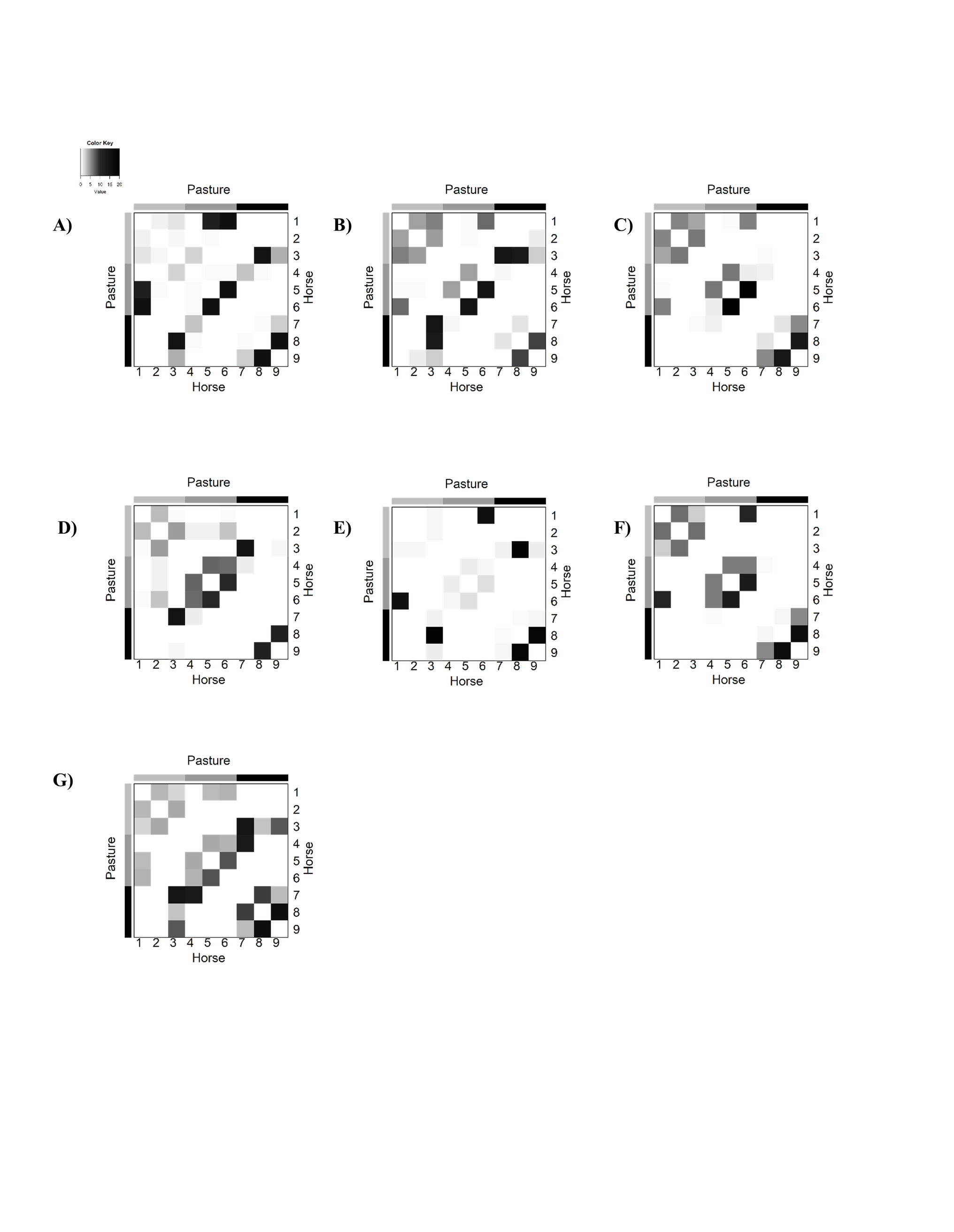

Supplement: S1 Fig — Darker cells represent longer durations (hours) of contact between horses. (TIF) [file pone.0210148.s002.tif]
