# Supplementary material for: Validation of modified radio-frequency identification tag firmware, using an equine population case study
Source: PLoS One. 2019 Jan 9;14(1):e0210148. doi: 10.1371/journal.pone.0210148 (PMC6326514; doi:10.1371/journal.pone.0210148)
Supplement: S1 Table — The tag that was located on the fence of pasture 4 was only activated on days 4 and 5 of the study. However, the contact duration on day 5 of the study was negligible, indicating that the tag was activated as horses walked passed the pasture. Many of the contact events were shorter in duration than what would be expected. This can be explained by the cold outdoor temperatures. (DOCX) [file pone.0210148.s003.docx]

| **Contact event duration (hours)** | |  | **Horse** | | | | | | | | |
| --- | --- | --- | --- | --- | --- | --- | --- | --- | --- | --- | --- |
|  | |  | **1** | **2** | **3** | **4** | **5** | **6** | **7** | **8** | **9** |
| **Day 1** | **Pasture** | **1** |  |  |  |  |  |  |  |  |  |
|  |  | **2** | 2.15 |  |  | 0.96 | 0.00 |  |  | 0.19 |  |
|  |  | **3** |  |  |  |  |  | 0.06 | 0.21 |  | <0.00 |
|  |  | **4** |  |  |  |  |  |  |  |  |  |
| **Day 4** | **Pasture** | **1** |  | 1.20 | 5.20 |  |  | 0.67 |  |  |  |
|  |  | **2** | <0.00 | <0.00 |  |  |  |  |  | 5.80 |  |
|  |  | **3** | <0.00 |  |  |  | 5.60 |  | 5.30 |  |  |
|  |  | **4** | <0.00 |  | 1.14 | 0.97 |  | 1.10 |  | 1.20 |  |
| **Day 5** | **Pasture** | **1** |  | 0.61 |  |  | <0.00 | 1.16 | 0.03 |  |  |
|  |  | **2** | <0.00 |  |  | 0.18 | <0.00 |  |  | 0.67 |  |
|  |  | **3** |  |  |  |  | 0.50 |  | 0.66 | 0.04 |  |
|  |  | **4** | <0.00 |  |  |  |  |  |  | <0.00 |  |
